# The effect of increasing the coinsurance rate on outpatient utilization of healthcare services in South Korea

**DOI:** 10.1186/s12913-017-2076-8

**Published:** 2017-02-20

**Authors:** Hyo Jung Lee, Sung-In Jang, Eun-Cheol Park

**Affiliations:** 1 Department of Public Health, Graduate School, Yonsei University, Seoul, Republic of Korea; 2 Institute of Health Services Research, Yonsei University College of Medicine, 50 Yonsei-ro, Seodaemun-gu, Seoul, 120-752 Republic of Korea; 3 Department of Preventive Medicine, Yonsei University College of Medicine, Seoul, Republic of Korea

**Keywords:** Increased coinsurance rate, Prescription drug cost policy, Healthcare utilization, Outpatient visit, Medical costs

## Abstract

**Background:**

The Korean healthcare system is composed of costly and inefficient structures that fail to adequately divide the functions and roles of medical care organizations. To resolve this matter, the government reformed the cost-sharing policy in November of 2011 for the management of outpatients visiting general or tertiary hospitals with comparatively mild diseases. The purpose of the present study was to examine the impact of increasing the coinsurance rate of prescription drug costs for 52 mild diseases at general or tertiary hospitals on outpatient healthcare service utilization.

**Methods:**

The present study used health insurance claim data collected from 2010 to 2013. The study population consisted of 505,691 outpatients and was defined as those aged 20–64 years who had visited medical care organizations for the treatment of 52 diseases both before and after the program began. To examine the effect of the cost-sharing policy on outpatient healthcare service utilization (percentage of general or tertiary hospital utilization, number of outpatient visits, and outpatient medical costs), a segmented regression analysis was performed.

**Results:**

After the policy to increase the coinsurance rate on prescription drug costs was implemented, the number of outpatient visits at general or tertiary hospitals decreased (β = −0.0114, *p* < 0.0001); however, the number increased at hospitals and clinics (β = 0.0580, *p* < 0.0001). Eventually, the number of outpatient visits to hospitals and clinics began to decrease after policy initiation (β = −0.0018, *p* < 0.0001). Outpatient medical costs decreased for both medical care organizations (general or tertiary hospitals: β = −2913.4, *P* < 0.0001; hospitals or clinics: β = −591.35, *p* < 0.0001), and this decreasing trend continued with time.

**Conclusions:**

It is not clear that decreased utilization of general or tertiary hospitals has transferred to that of clinics or hospitals due to the increased cost-sharing policy of prescription drug costs. This result indicates the cost-sharing policy, intended to change patient behaviors for healthcare service utilization, has had limited effects on rebuilding the healthcare system and the function of medical care organizations.

**Electronic supplementary material:**

The online version of this article (doi:10.1186/s12913-017-2076-8) contains supplementary material, which is available to authorized users.

## Background

Since South Korea established a national health insurance program in 1989, health expenditures and accessibility to healthcare and medical needs have rapidly increased [1]. In South Korea, total health expenditures accounted for 7.6% of gross domestic production in 2012 [2]. This total health expenditure is lower than the Organization for Economic Co-operation and Development (OECD) average of 9.3%. However, the growth rate in health expenditure per person from 2001 to 2011 is higher than that of most OECD countries (South Korea: 9.3%; OECD average: 4.0%). Thus, achieving control over the constantly increasing health expenditure has become a key healthcare reform concern in South Korea [3].

There have been various discussions surrounding the financial stability of health insurance in South Korea. One of the proposed strategies is to build a sustainable healthcare system. To improve healthcare systems, simultaneous pursuit of three aims—improving the experience of care, improving the health of populations, and reducing per capita costs of health care—is required. The role of the South Korean government, as an integrator that accepts responsibility for these three aims, includes the redesign of primary care, population health management, and financial management [4]. In particular, the South Korean government attempted to improve primary health care and manage finances efficiently by assigning an appropriate role for medical care organizations according to size and function [5]. In South Korea, medical care is divided between clinical and hospital organizations by function, and hospitals are further divided into specialist and general hospitals according to structural characteristics. Medical law defines clinics (less than 30 beds) as centers treating outpatients and hospitals (more than 30 beds) as treating inpatients. General hospitals have more than 100 beds and at least 7 medical departments, including essential medical departments designated by medical law. Additionally, the Minister of Health and Welfare is able to specify tertiary hospitals as more specialist hospitals treating severe diseases compared to general hospitals with several requirements such as manpower, facilities, and equipment.

However, despite the use of this classification, individuals are able to choose any medical care organizations, from clinics in their community to hospitals [6]. Thus, the South Korean healthcare system contains inefficient structures that fail to adequately divide the functions and roles of medical care organizations. Accordingly, their functions overlap and all medical care organizations compete with each other, regardless of hospital type [7]. In addition, patients are focused on general hospitals in metropolitan areas despite having mild diseases and have to pay more for hospital services compared to clinic services. When evaluating diagnostic codes of outpatients according to hospital type, hypertension, diabetes mellitus, and acute upper respiratory tract infections, which are treatable in the primary healthcare setting, are the most frequent diseases treated in all hospital types. In addition, 44 tertiary hospitals (0.07% of medical care organizations) account for 23% of health insurance expenditure and this percentage is increasing [8]. Thus, in October 2011, the government reformed the policy for the management of outpatients visiting general hospitals for the care of comparatively mild diseases. This policy resulted in an increase in the existing 30% of coinsurance rate on prescription drug costs for 52 types of diseases to 50% in tertiary hospitals and 40% in general hospitals [5, 9].

In general, cost sharing, including coinsurance, copayment, and deductible, refers to any financing arrangement where the cost of the services used is supported in part by the user. The main objective is to prevent unnecessary utilization of health services and to stabilize insurance finances [10]. A further objective is to shift health care expenditures from public to private resources and secure additional finances to sustain the functioning of health services [11]. There have been many previous studies on cost sharing. The findings of the RAND Health Insurance Experiment and other studies of non-elderly insured populations reported that cost sharing reduced total health care spending and utilization without harming the health of individuals [12, 13]. However, some studies have reported higher cost sharing to be associated with adverse outcomes, particularly among vulnerable populations such as elderly and poor patients [14–16]. In studies that were not limited to patients with certain chronic illnesses, increased cost sharing was not found to be associated with increased number of outpatient visits, emergency department visits, or hospitalizations [17].

Previous research on cost sharing performed by South Korea found that low-income patients were more sensitive to cost sharing than high-income patients, and users of general hospitals were less sensitive to cost sharing than the users of clinics [18]. Furthermore, another study suggested that cost sharing among the elderly had little effect on controlling health care utilization [10]. However, some studies have demonstrated that cost sharing decreases medical costs and visit days per outpatient [19, 20]. The results of studies regarding the effect of increasing the coinsurance rate of prescription drug costs—our policy of interest—were inconsistent [9, 21, 22]. In addition, few studies have considered individual characteristics, such as sex, age, and income, even where individual characteristics are important factors for healthcare utilization, particularly in South Korea due to free choice of medical care organizations and a payment system based on fee-for-service. Therefore, the purpose of the present study was to examine the impact of changing the coinsurance rate of prescription drug costs for 52 mild diseases on outpatient healthcare service utilization using nationally representative data.

## Methods

### Study population

The present study used National Sample Cohort data, including all medical claims, from 2010 to 2013 released by the National Health Insurance Service (NHIS), which consists of details of patient healthcare utilization. The data included approximately 100 million people sampled by sex, age, employment status (employed or self-employed), income, and individual total medical costs. Our study population was defined as outpatients aged 20–64 years who had visited medical care organizations more than once, both before and after the policy change. for the treatment of 52 diseases, including acute bronchitis, gastritis, duodenitis, and hypertension. These 52 diseases are classified according to the International Classification of Diseases groupings (ICD-10) and details regarding each type and its description are presented in the Additional file 1. Additionally, the present study included only National Health Insurance (NHI) beneficiaries who were enrolled in health insurance provided by public sector. Health insurance in South Korea is classified into either NHI or Medical Aid. Individuals whose single-family household income is less than $600 per month qualify for Medical Aid while others should join NHI. Since NHI and Medical Aid have slightly differing copayment systems, we included NHI beneficiaries only. The present study was approved by the Institutional Review Board, Yonsei University Graduate School of Public Health (2014-239). The requirement for informed consent from patients was waived as patient information was anonymized prior to the study analysis.

### Measures

We used the proportion of general or tertiary hospital utilization, number of outpatient visits, and medical costs as dependent variables to reflect the shift of outpatients into hospitals or clinics from general or tertiary hospitals. All dependent variables were calculated in units of person–month. General or tertiary hospital utilization was defined as the proportion of general or tertiary hospital utilization among total healthcare utilization. The proportion of general or tertiary hospital utilization per month was calculated as (the number of outpatient visits into general or tertiary hospitals per person–month/the number of outpatient visits into total healthcare utilization per person–month) × 100. The numbers of outpatient visits and medical costs per person–month were analyzed by categorizing costs into general or tertiary hospital and hospital or clinic. Medical costs indicated the total costs of visiting physicians and prescription drugs. The monetary unit of medical costs was KRW, with 1000 KRW corresponding to approximately 1 US$.

For the analysis of the relationship between the introduction of the policy and healthcare utilization, we adjusted for individual characteristics. Individual characteristics included age, sex, income, residence region, Charlson Comorbidity Index (CCI), and all cause admission during the previous year. Demographic factors, including age, sex, income, and residence region, are known to be associated with health care utilization [23–25]. Further, health-related factors such as CCI and recently history of admission may affect the pattern of health care utilization [26–28]. Age in years was classified into five groups as follows; 20–29, 30–39, 40–49, 50–59, and 60–64. Regions were categorized into urban and rural. Income level was estimated using the average monthly health insurance premium. Individuals with NHI provided by their employer paid a monthly insurance premium according to annual salary, and those who were self-employed paid a premium according to their property value. Low-income was defined as the bottom 20 percentiles of health insurance premiums, middle-income was defined as the 20–80 percentiles of the premiums, and high-income was defined as the top 20 percentiles of premiums. The CCI was used to account for the effects of comorbid disorders or diseases. CCI was calculated monthly according to Quan’s methods [29]. Nineteen diseases were classified into scores of 1, 2, 3, and 6 [30]. The CCI was calculated from the sum of all scores and given extra scores in accordance with age. In the present study, CCI was grouped as scores of 0, 1, 2, and 3 or over.

### Statistical analysis

We examined the distribution of individual characteristics by analyzing their frequencies. Student’s *t*-test was performed for dependent variables, proportion of general or tertiary hospital utilization, number of outpatient visits, and medical costs both before and after the introduction of the program. Segmented regression analysis of interrupted time series analysis was used to examine policy effects [31]. Our segmented regression analysis equation was:

$$ \begin{array}{c}\hfill {Y}_{it}={\beta}_0+{\beta}_1\times tim{e}_t+{\beta}_2\times 2011\kern0.5em  policy+{\beta}_3\times tim e\kern0.5em  after\kern0.5em  2011\kern0.5em  policy\kern0.5em +\hfill \\ {}\hfill {\beta}_4\times seaso{n}_t+{X}_{it}+{e}_{it}\hfill \end{array} $$

*Y*: dependent variables
*i*: each variables
*t*: time period
*time*: a continuous variable beginning in January 2010
*2011 policy*: changing coinsurance rate on prescription drug cost in November 2011, a binary variable (0 before; 1 after)
*time after 2011 policy*: a continuous variable beginning in November 2011
*season*: seasonality (spring, summer, fall, winter)
*X*: independent variables
*e*: error term

In the present study analysis, the 2011 policy began in November 2011, as there was a 1-month-lagged effect after implementation of the policy. For the segmented regression analysis, the Generalized Estimation Equation (GEE) was used. PROC GENMOD was performed for the GEE with link identity, normal distribution, and type = AR (1). Repeated measures were considered and the unit of analysis was person-month. Subgroup analyses by income and sex were also performed. All statistical analyses were performed using SAS statistical software version 9.2. All calculated *p*-values were two-sided and considered statistically significant at *p* < 0.05.

## Results

Table 1 shows the general characteristics of the study population. A total of 505,691 outpatients were included in the analysis. The highest proportion was in the 50–59 years old group at 131,556 (26.0%). There were 230,371 (45.6%) men and 275,320 (54.4%) women. More than half (59.7%) were middle-income earners, lived in an urban area (71.2%), and had 0 points on the CCI (64.4%). The majority of the study population had no disability (96.4%). A total of 48,922 (9.7%) outpatients were admitted during the previous year. Based on the number of outpatients, the most common disease was acute bronchitis, unspecified (ICD-10: J20.9, 335,686 outpatients, 66.4%). When viewed in terms of the total numbers of visits, patients with essential hypertension (ICD-10: I10) had the most visits to medical care organizations (1,638,083 cases, 13.6%).

**Table 1:** General characteristics of the study population

	Number	Percent
Age		
20–29	49,609	9.8
30–39	112,854	22.3
40–49	131,077	25.9
50–59	131,556	26.0
60–64	80,595	15.9
Sex		
Men	230,371	45.6
Women	275,320	54.4
Income		
Low (0–20%)	70,648	14.0
Middle (20.1–80%)	302,061	59.7
High (80.1–100%)	132,982	26.3
Region		
Urban	359,964	71.2
Rural	145,727	28.8
Charlson comorbidity index		
0	325,786	64.4
1	126,791	25.1
2	41,673	8.2
3+	11,441	2.3
Disability		
Normal	487,371	96.4
Mild disability	15,519	3.1
Severe disability	2,801	0.6
All cause admission at previous year		
Non-admission	456,769	90.3
Admission	48,922	9.7
Most frequent disease (ICD-10 code)		
Acute bronchitis, unspecified (J20.9)	335,686	66.4
Acute tonsillitis (J03.0 ~ J03.9)	201,650	39.9
Gastritis and duodenitis (K29.0 ~ K29.9)	192,444	38.1
Acute upper respiratory infections of multiple and unspecified sites (J06.0 ~ J06.9)	173,381	34.3
Allergic contact dermatitis due to other agents or unspecified cause (L23.8, L23.9)	140,817	27.8
Total	505,691	100.0

**Fig. 1:**
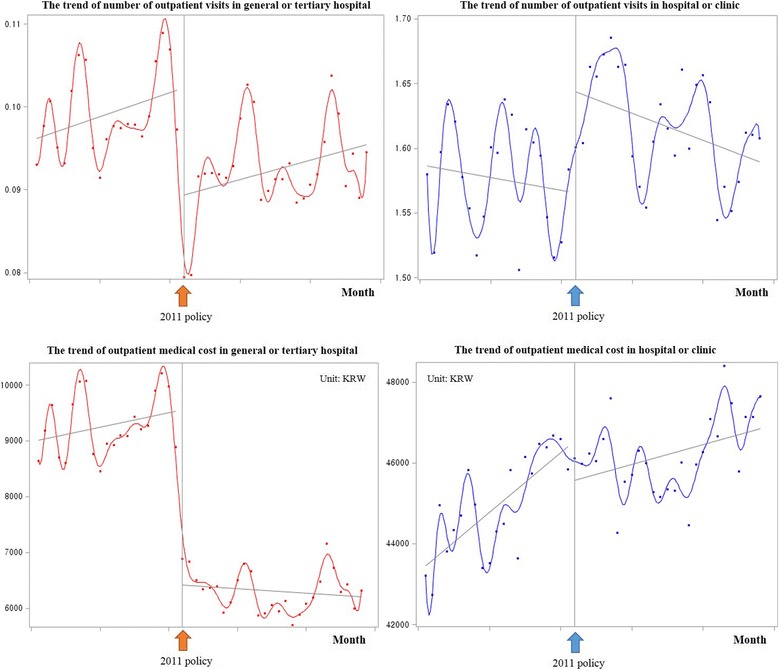
The trends of each dependent variable for month

The trends of each dependent variable before and after the policy are shown in Table 2. The proportion of general or tertiary hospital utilization was 5.9% before the 2011 policy and 5.4% after the 2011 policy. The number of outpatient visits decreased in terms of general or tertiary hospital utilization per month but increased in hospital or clinic utilization after the 2011 policy (general or tertiary hospital utilization: 0.099 - >0.092, *p* < 0.0001; hospital or clinic utilization: 1.576 - >1.617, *p* < 0.0001). Outpatient medical costs also decreased for general or tertiary hospital utilization per month but increased in hospital or clinic utilization after the 2011 policy (general or tertiary hospital utilization: 9273.8 - >6316.4, *p* < 0.0001; hospital or clinic utilization: 44,935.1 - >46,206.1, *p* < 0.0001). The trends of each dependent variable per month are shown in Fig. 1.

**Table 2:** The trends of each dependent variable before and after the 2011 policy. Unit: Mean ± SD

	Before intervention	After intervention	*p*-value
	2010.1–2011.9	2011.10–2013.12	
Percentage of general and tertiary hospital utilization	5.921 ± 0.390	5.410 ± 0.380	<0.0001
General and tertiary hospital utilization			
Number of outpatient visits	0.099 ± 0.005	0.092 ± 0.006	<0.0001
Outpatient medical costs	9273.8 ± 542.8	6316.4 ± 366.5	<0.0001
Hospital and clinic utilization			
Number of outpatient visits	1.576 ± 0.042	1.617 ± 0.041	<0.0001
Outpatient medical costs	44935.1 ± 1242.6	46206.1 ± 976.2	<0.0001

Table 3 shows the results of the segmented regression analysis. The 2011 policy decreased the percentage of general or tertiary hospital utilization (β = −1.6184, *p* < 0.0001). After the 2011 policy, the number of outpatient visits decreased for general or tertiary hospitals (β = −0.0114, *p* < 0.0001) and increased for hospitals or clinics (β = 0.0580, *p* < 0.0001). However, the number of outpatient visits in hospitals or clinics exhibited a downward trend after the policy. Outpatient medical costs decreased in both medical care organizations (general or tertiary hospitals: β = −2913.4, *p* < 0.0001; and hospital or clinic utilization: β = −591.35, *p* <0.0001). This trend continued to decrease over time. The percentage of general or tertiary hospital utilization by outpatients with acute bronchitis recovered (*β* = 0.0167, *p* = 0.0010), while that by outpatients with essential hypertension decreased (β = −0.0154, *p* = 0.0029) over time after the 2011 policy began.

**Table 3:** The results of the segmented regression analysis

	Time		2011 policy		Time after 2011 policy	
	Estimate*	*p*-value	Estimate*	*p*-value	Estimate*	*p*-value
Total						
Percentage of general or tertiary hospital utilization	−0.0044	0.0658	−1.6184	<0.0001	0.0036	0.1786
General and tertiary hospital utilization						
Number of outpatient visits	0.0001	0.0034	−0.0114	<0.0001	0.0001	0.3159
Outpatient medical costs	1.3140	0.7452	−2913.4	<0.0001	−23.684	<0.0001
Hospitals and clinic utilization						
Number of outpatient visits	0.0001	0.9998	0.0580	<0.0001	−0.0018	<0.0001
Outpatient medical costs	106.41	<0.0001	−591.35	<0.0001	−97.722	<0.0001
Acute bronchitis, unspecified (J20.9)						
Percentage of general and tertiary hospital utilization	−0.0086	0.0500	−0.6044	<0.0001	0.0167	0.0010
General and tertiary hospital utilization						
Number of outpatient visits	0.0001	0.5675	−0.0060	<0.0001	0.0001	0.0983
Outpatient medical costs	6.5779	0.3676	−1144.6	<0.0001	−9.5789	0.2411
Hospital and clinic utilization						
Number of outpatient visits	0.0023	<0.0001	0.0110	0.0481	−0.0041	<0.0001
Outpatient medical costs	127.14	<0.0001	−1057.7	<0.0001	−123.28	<0.0001
Essential hypertension (I10.0, I10.9)						
Percentage of general and tertiary hospital utilization	−0.0164	0.0001	−1.0330	<0.0001	−0.0154	0.0029
General and tertiary hospital utilization						
Number of outpatient visits	−0.0001	0.1233	−0.0095	<0.0001	−0.0003	<0.0001
Outpatient medical costs	−14.902	0.0543	−2381.0	<0.0001	−51.712	<0.0001
Hospital and clinic utilization						
Number of outpatient visits	0.0002	0.3478	0.0363	<0.0001	−0.0014	<0.0001
Outpatient medical costs	182.60	<0.0001	−877.34	<0.0001	−202.80	<0.0001

*Adjusted for age, sex, income, region, CCI, disability, and all cause admission during the previous year

After the policy began, the increasing coinsurance rate, number of outpatient visits, and outpatient medical costs in both medical care organizations demonstrated the same trend in the total population regardless of income and sex (Table 4). General or tertiary hospital utilization, including both the number of outpatient visits and outpatient medical costs, decreased; however, hospital or clinic utilization did not increase to the same extent as the decrease in general or tertiary hospital utilization. Rather, outpatient medical costs decreased. Unlike the results of the subgroup analysis by income, we identified that the absolute values of the coefficient for time after the 2011 policy were higher in women than in men, indicating women were more likely to be sensitive to the 2011 policy.

**Table 4:** The results of the segmented regression analysis by income

	Time		2011 policy		Time after 2011 policy	
	Estimate	*p*-value	Estimate	*p*-value	Estimate	*p*-value
Low-income*						
Percentage of general or tertiary hospital utilization	0.0045	0.4908	−1.1350	<0.0001	−0.0066	0.4063
General or tertiary hospital utilization						
Number of outpatient visits	0.0002	0.0156	−0.0067	<0.0001	−0.0001	0.5488
Outpatient medical costs	17.348	0.1097	−2146.8	<0.0001	−38.488	0.0026
Hospital or clinic utilization						
Number of outpatient visits	0.0001	0.3191	0.054	<0.0001	−0.0024	<0.0001
Outpatient medical costs	113.3	<0.0001	−516.58	0.0111	−116.55	<0.0001
Middle-income*						
Percentage of general or tertiary hospital utilization	0.0008	0.8077	−1.2203	<0.0001	−0.0014	0.7231
General or tertiary hospital utilization						
Number of outpatient visits	0.0002	<0.0001	−0.0079	<0.0001	0.0001	0.6918
Outpatient medical costs	12.213	0.0173	−2349.3	<0.0001	−32.848	<0.0001
Hospital or clinic utilization						
Number of outpatient visits	0.0002	0.3565	0.0490	<0.0001	−0.0018	<0.0001
Outpatient medical costs	110.11	<0.0001	−980.52	<0.0001	−93.052	<0.0001
High-income*						
Percentage of general or tertiary hospital utilization	−0.0163	0.0017	−1.4079	<0.0001	0.0059	0.3481
General or tertiary hospital utilization						
Number of outpatient visits	0.0001	0.6515	−0.0070	<0.0001	0.0001	0.8488
Outpatient medical costs	1.4560	0.8684	−2873.7	<0.0001	−40.050	0.0001
Hospital or clinic utilization						
Number of outpatient visits	−0.0002	0.5677	0.0576	<0.0001	−0.0013	0.0003
Outpatient medical costs	103.51	<0.0001	−480.34	0.0003	−106.28	<0.0001
Men^+^						
Percentage of general or tertiary hospital utilization	−0.0041	0.3630	−1.1856	<0.0001	0.0135	0.0182
General or tertiary hospital utilization						
Number of outpatient visits	0.0004	<0.0001	−0.0056	<0.0001	−0.0002	0.0824
Outpatient medical costs	331.91	<0.0001	−2029.9	<0.0001	−441.73	<0.0001
Hospital or clinic utilization						
Number of outpatient visits	0.0044	<0.0001	0.0241	<0.0001	−0.0036	<0.0001
Outpatient medical costs	278.33	<0.0001	−306.79	0.0224	−313.60	<0.0001
Women^+^						
Percentage of general or tertiary hospital utilization	−0.0110	0.0005	−0.9660	<0.0001	0.0362	<0.0001
General or tertiary hospital utilization						
Number of outpatient visits	0.0003	<0.0001	−0.0041	<0.0001	0.0001	0.8235
Outpatient medical costs	358.99	<0.0001	−1220.7	<0.0001	−536.60	<0.0001
Hospital or clinic utilization						
Number of outpatient visits	0.0070	<0.0001	0.0311	<0.0001	−0.0096	<0.0001
Outpatient medical costs	352.36	<0.0001	−318.52	0.2383	−507.25	<0.0001

*Adjusted for age, sex, region, CCI, disability, and all cause admission at previous year

^+^Adjusted for age, income, region, CCI, disability, and all cause admission at previous year

## Discussion

Medical costs per visit for the same diagnostic code are higher in larger hospitals and are 3–4 times higher in tertiary hospitals than in clinics [8]. Thus, if outpatients are concentrated in tertiary hospitals, health insurance finances become an economic burden. In addition, since patients are less likely to visit clinics and small hospitals, the quality of care in clinics and small hospitals is reduced, which may lead to patient distrust of clinics and small hospitals. Patients will be then much less likely to visit clinics and small hospitals. Once this vicious cycle is repeated, financial difficulties, both in health insurance and in clinics or small hospitals, will further increase [32]. Tertiary hospitals may not be able to adequately perform their primary function of treating severe diseases. Accordingly, outpatients presenting for 52 mild diseases paying prescription drug costs differently depending on the hospital type represents a potential method of resolving the above matters.

The findings of the present study indicate that changing the coinsurance rate on prescription drug costs was associated with changes in outpatient healthcare service utilization. The introduction of the 2011 policy decreased the number of outpatient visits in general or tertiary hospitals. The number of outpatient visits in hospitals or clinics increased with the introduction of the 2011 policy; however, it decreased over time after the 2011 policy. In addition, outpatient medical costs decreased in both general or tertiary hospitals and hospitals or clinics. Therefore, the 2011 policy, changing the coinsurance rate on prescription drug costs, partially shifted visits from general hospitals or tertiary hospitals to clinics.

Studies have reported that cost sharing reduces the needs of various health services and the burden of health insurance [17]. The reducing rate of outpatient medical cost increases for hospitals or clinics, despite the increase in the number of outpatient visits immediately after the 2011 policy, may be attributable to decreased pharmaceutical use, which could be caused by reduced unnecessary prescription drug treatment in the treatment intensity, although the decision making process on treatment intensity needs to be examined more carefully [33]. However, the effect of cost sharing is likely to involve side effects as our results demonstrate that the number of outpatient visits for general or tertiary hospitals tended to increase while that for hospitals or clinics decreased over time after the 2011 policy [21]. There are several possible explanations for these results. First, increasing the coinsurance rate from 10 to 20% may have been insufficient to prevent patients from excess visits to general or tertiary hospitals, and the effect of the cost-sharing policy may not have been maintained for a substantial period of time [34]. Second, increased cost sharing may be related to adverse events such as hospitalization and worsening clinical outcomes due to the decline in access to general or tertiary hospitals [33, 35]. Thus, the increase in hospitalization may have led to a decrease in outpatients, causing the number of outpatient visits for hospitals or clinics to decrease over time. Additionally, the present study demonstrates that both the number of outpatient visits and medical costs associated with hypertension decreased compared to other diseases after the 2011 policy in all medical care organizations. This observation may be explained in two ways. First, the policy increases the coinsurance rate for patients with specific diagnostic codes. Thus, outpatients with hypertension are able to continue visiting general and tertiary hospitals using other diagnostic codes such as for hypertensive heart disease (ICD-10: I11) rather than essential hypertension (ICD-10: I10). Alternatively, it is possible that hospitalization for hypertension increased over the study period [21].

We examined the effect of the 2011 policy on healthcare utilization according to income and sex via subgroup analysis. Income is an important factor for cost-sharing policies. We observed that women were more likely to be sensitive to the 2011 policy since the absolute values of the coefficient for time after the implementation of the 2011 policy were higher in women than in men. This result was consistent with those of previous studies [36, 37]. However, our study did not identify any direct evidence for a difference in healthcare utilization according to income level, despite previous reports that patients with lower income are more sensitive to the cost-sharing policy [18, 38, 39]. In the present study, the increased coinsurance rate did not have a consistent effect difference based on income. Thus, we were unable to evaluate price elasticity or moral hazards.

As a result, the 2011 policy did not control healthcare utilization and health insurance finance in the long term. It is important to observe the effect of increasing the coinsurance rate on prescription drug costs for 52 diseases in the future. In addition, establishment of the criteria for determining the primary diagnosis to prevent using another diagnostic code and follow-up investigations for continuously monitoring patients and hospitals are needed.

The present study has some strengths compared to previous studies. First, we used data from a nationally representative large sample size, which reflect the overall medical information of South Koreans. Such data are especially helpful in establishing evidence-based health policies. Second, to our knowledge, there are few previous studies from South Korea that have analyzed the correlations between policy introduction and healthcare utilization with consideration of individual characteristics. Although some studies have assessed policy effects, they have used the total sum of outpatient visits and medical costs per month rather than per person-month. Thus, we are able to provide more detailed information on the policy related to the coinsurance rate of prescription drug costs.

The present study also had some limitations related to limited data and methodology issues. First, there may have been other external factors, not considered in our study, which affected healthcare utilization. For example, in the case of hypertension, the South Korean government reformed the prices of existing drugs in April 2012 and revised guidelines restricting prescription for antihypertensive drugs in January 2013. In addition, some individuals, irrespective of the coinsurance rate, prefer general or tertiary hospitals over clinics. Thus, our results require careful interpretation. Further, since the most frequently treated diseases of the 52 are influenced by seasonal, socio-economic and demographic characteristics, and personal health status, healthcare utilization may have been affected [18, 40, 41]. Furthermore, detailed covariates related with each disease were not adjusted as we analyzed all 52 diseases, which included chronic diseases, as well as acute diseases. As each disease has different characteristics, other covariates may be needed. Furthermore, we did not assess the severity of disease as information related to this assessment was not available in the present study. However, we considered severity (in terms of CCI) for a more detailed study. Last, hospital characteristics, including the quality of the offered services, were not captured in our study, and there may be hospital effects such as quality on healthcare utilization.

## Conclusions

Our findings demonstrate that the introduction of the 2011 policy increasing the coinsurance rate on prescription drug costs decreased utilization of outpatient visits in general or tertiary hospitals. However, outpatient medical costs decreased in all medical care organizations. As we did not consider other external factors related to healthcare utilization in our analysis, it is not clear whether decreased utilization of general or tertiary hospitals transferred to demand for clinics or hospitals care of the 2011 policy. This result indicates that the price policy intended to change behaviors for healthcare service utilization had limited effects on rebuilding the healthcare system or the function of medical care organizations.

## Additional file

Additional file 1: 52 diseases to apply the cost-sharing policy. (DOC 119 kb)

## Abbreviations

CCI: Charlson Comorbidity Index
GEE: Generalized estimation equation
ICD: International Classification of Diseases groupings
NHI: National Health Insurance
NHIS: National Health Insurance Service
OECD: Organization for Economic Cooperation and Development
