# Supplementary material for: The effect of increasing the coinsurance rate on outpatient utilization of healthcare services in South Korea
Source: BMC Health Serv Res. 2017 Feb 20;17:152. doi: 10.1186/s12913-017-2076-8 (PMC5319163; doi:10.1186/s12913-017-2076-8)
Supplement: Additional file 1: — 52 diseases to apply the cost-sharing policy. (DOC 119 kb) [file 12913_2017_2076_MOESM1_ESM.doc]

**Additional file 1. 52 diseases to apply the cost-sharing policy.**

| **Type** | **Disease (ICD-10)** |
| --- | --- |
| 1 | Other gastroenteritis and colitis of infectious and unspecified origin (A09.0∼A09.9) |
| 2 | Tinea manuum (B35.2) |
|  | Tinea pedis (B35.3) |
|  | Tinea corporis (B35.4) |
|  | Tinea imbricata (B35.5) |
|  | Tinea inguinalis (B35.6) |
|  | Other dermatophytoses (B35.8) |
|  | Dermatophytosis, unspecified (B35.9) |
| 3* | Type 2 diabetes mellitus with renal complications (E11.2) |
|  | Type 2 diabetes mellitus with ophthalmic complications (E11.3) |
|  | Type 2 diabetes mellitus with neurological complications (E11.4) |
|  | Type 2 diabetes mellitus with peripheral circulatory complications (E11.5) |
|  | Type 2 diabetes mellitus with other specified complications (E11.6) |
|  | Type 2 diabetes mellitus with multiple complications (E11.7) |
|  | Type 2 diabetes mellitus with unspecified complications (E11.8) |
|  | Type 2 diabetes mellitus without complications (E11.9) |
| 4 | Disorders of lipoprotein metabolism and other lipidaemias (E78.0∼E78.9) |
| 5 | Hordeolum and chalazion (H00.0∼H00.1) |
| 6 | Disorders of lacrimal system (H04.0∼H04.9) |
| 7 | Conjunctivitis (H10.0∼H10.9) |
| 8 | Senile cataract (H25.0∼H25.9) |
| 9 | Disorders of refraction and accommodation (H52.0∼H52.7) |
| 10 | Cellulitis of external ear (H60.1) |
|  | Other infective otitis externa (H60.3) |
|  | Acute otitis externa, noninfective (H60.5) |
|  | Other otitis externa (H60.8) |
|  | Otitis externa, unspecified (H60.9) |
| 11 | Essential (primary) hypertension (I10.0) |
|  | Hypertension, unspecified (I10.9) |
| 12 | Acute nasopharyngitis [common cold] (J00) |
| 13 | Acute sinusitis (J01.0∼J01.9) |
| 14 | Acute pharyngitis (J02.0∼J02.9) |
| 15 | Acute tonsillitis (J03.0∼J03.9) |
| 16 | Acute laryngitis and tracheitis (J04.0∼J04.2) |
| 17 | Acute upper respiratory infections of multiple and unspecified sites (J06.0∼J06.9) |
| 18 | Acute bronchitis, unspecified (J20.9) |
| 19 | Vasomotor and allergic rhinitis (J30.0∼J30.4) |
| 20 | Chronic nasopharyngitis (J31.1) |
|  | Chronic pharyngitis (J31.2) |
| 21 | Chronic sinusitis (J32.0∼J32.9) |
| 22 | Asthma (J45.0∼J45.9) |
| 23 | Gastro-oesophageal reflux disease (K21.0∼K21.9) |
| 24 | Gastric ulcer : acute without haemorrhage or perforation (K25.3) |
|  | Gastric ulcer : chronic without haemorrhage or perforation (K25.7) |
|  | Gastric ulcer : unspecified as acute or chronic, without haemorrhage or perforation (K25.9) |
| 25 | Peptic ulcer, site unspecified : acute without haemorrhage or perforation (K27.3) |
|  | Peptic ulcer, site unspecified : chronic without haemorrhage or perforation (K27.7) |
|  | Peptic ulcer, site unspecified : unspecified as acute or chronic, without haemorrhage or perforation (K27.9) |
| 26 | Gastritis and duodenitis (K29.0∼K29.9) |
| 27 | Functional dyspepsia (K30) |
| 28 | Allergic and dietetic gastroenteritis and colitis (K52.2) |
|  | Indeterminate colitis (K52.3) |
|  | Other specified noninfective gastroenteritis and colitis (K52.8) |
|  | Noninfective gastroenteritis and colitis, unspecified (K52.9) |
| 29 | Irritable bowel syndrome (K58.0∼K58.9) |
| 30 | Constipation (K59.0) |
|  | Functional diarrhoea (K59.1) |
|  | Neurogenic bowel, not elsewhere classified (K59.2) |
|  | Anal spasm (K59.4) |
|  | Other specified functional intestinal disorders (K59.8) |
|  | Functional intestinal disorder, unspecified (K59.9) |
| 31 | Fatty (change of) liver, not elsewhere classified (K76.0) |
|  | Liver disease, unspecified (K76.9) |
| 32 | Other atopic dermatitis (L20.8) |
|  | Atopic dermatitis, unspecified (L20.9) |
| 33 | Allergic contact dermatitis due to other agents (L23.8) |
|  | Allergic contact dermatitis, unspecified cause (L23.9) |
| 34 | Urticaria (L50.0∼L50.9) |
| 35 | Other arthritis (M13.0∼M13.9) |
| 36 | Other spondylosis (M47.8) |
|  | Spondylosis, unspecified (M47.9) |
| 37 | Cervical disc disorder, unspecified (M50.9) |
| 38 | Other specified intervertebral disc degeneration (M51.3) |
|  | Schmorl nodes (M51.4) |
|  | Other specified intervertebral disc disorders (M51.8) |
|  | Intervertebral disc disorder, unspecified (M51.9) |
| 39 | Other dorsalgia (M54.8) |
|  | Dorsalgia, unspecified (M54.9) |
| 40 | Calcific tendinitis (M65.2) |
|  | Trigger finger (M65.3) |
|  | Other synovitis and tenosynovitis (M65.8) |
|  | Synovitis and tenosynovitis, unspecified (M65.9) |
| 41 | Adhesive capsulitis of shoulder (M75.0) |
|  | Bicipital tendinitis (M75.2) |
|  | Shoulder lesion, unspecified (M75.9) |
| 42 | Other enthesopathies, not elsewhere classified (M77.8) |
|  | Enthesopathy, unspecified (M77.9) |
| 43 | Myalgia (M79.1) |
|  | Hypertrophy of (infrapatellar) fat pad (M79.4) |
|  | Pain in limb (M79.6) |
|  | Other specified soft tissue disorders (M79.8) |
|  | Soft tissue disorder, unspecified (M79.9) |
| 44 | Osteoporosis without pathological fracture (M81.0∼M81.9) |
| 45 | Acute cystitis (N30.0) |
|  | Cystitis, unspecified (N30.9) |
| 46 | Chronic prostatitis (N41.1) |
| 47 | Acute vaginitis (N76.0) |
|  | Acute vulvitis (N76.2) |
| 48 | Menopausal and female climacteric states (N95.1) |
|  | Postmenopausal atrophic vaginitis (N95.2) |
|  | Menopausal and perimenopausal disorder, unspecified (N95.9) |
| 49 | Sprain and strain of lumbar spine (S33.5) |
|  | Sprain and strain of sacroiliac joint (S33.6) |
|  | Sprain and strain of other and unspecified parts of lumbar spine and pelvis (S33.7) |
| 50 | Sprain and strain of finger(s) (S63.6) |
|  | Sprain and strain of other and unspecified parts of hand (S63.7) |
| 51 | Sprain and strain of other and unspecified parts of knee (S83.6) |
| 52 | Sprain and strain of toe(s) (S93.5) |
|  | Sprain and strain of other and unspecified parts of foot (S93.6) |

*Except for patients receiving insulin treatment.
